# Inhibition of UBA52 induces autophagy via EMC6 to suppress hepatocellular carcinoma tumorigenesis and progression

**DOI:** 10.1111/jcmm.18164

**Published:** 2024-03-06

**Authors:** Li Tong, Xiaofei Zheng, Tianqi Wang, Wang Gu, Tingting Shen, Wenkang Yuan, Siyu Wang, Songlin Xing, Xiaoying Liu, Chong Zhang, Chao Zhang

**Affiliations:** ^1^ Department of General Surgery The First Affiliated Hospital of Anhui Medical University Hefei China; ^2^ Department of Pathology The First Affiliated Hospital of Anhui Medical University Hefei China; ^3^ College of Life Sciences of Anhui Medical University Hefei China

**Keywords:** autophagy, EMC6, hepatocellular carcinoma, progression, tumorigenesis, UBA52

## Abstract

Ubiquitin A‐52 residue ribosomal protein fusion product 1 (UBA52) has a role in the occurrence and development of tumours. However, the mechanism by which UBA52 regulates hepatocellular carcinoma (HCC) tumorigenesis and progression remains poorly understood. By using the Cell Counting Kit (CCK‐8), colony formation, wound healing and Transwell assays, we assessed the effects of UBA52 knockdown and overexpression on the proliferation and migration of HCC cells in vitro. By establishing subcutaneous and metastatic tumour models in nude mice, we evaluated the effects of UBA52 on HCC cell proliferation and migration in vivo. Through bioinformatic analysis of data from the Gene Expression Profiling Interactive Analysis (GEPIA) and The Cancer Genome Atlas (TCGA) databases, we discovered that UBA52 is associated with autophagy. In addition, we discovered that HCC tissues with high UBA52 expression had a poor prognosis in patients. Moreover, knockdown of UBA52 reduced HCC cell growth and metastasis both in vitro and in vivo. Mechanistically, knockdown of UBA52 induced autophagy through EMC6 in HCC cells. These findings suggest that UBA52 promoted the proliferation and migration of HCC cells through autophagy regulation via EMC6 and imply that UBA52 may be a viable novel treatment target for HCC patients.

## 1 INTRODUCTION

Hepatocellular carcinoma (HCC) is one of the most common malignant digestive tumours and is the third leading cause of cancer mortality worldwide.^1^, ^2^, ^3^ Cancer death rates have generally declined in recent decades, but the mortality of HCC has increased.^4^ Many etiological factors contribute to the development of HCC and include alcohol abuse, obesity, persistent infections with hepatitis B or C viruses, aflatoxin exposure and fatty liver disease.^5^ Although the diagnostic and therapeutic methods for HCC have been improved, the prognosis of patients with recurrence or metastasis remains poor.^6^ Unfortunately, the molecular mechanisms underlying HCC recurrence and metastasis are incompletely elucidated.^7^ Understanding the molecular mechanisms of HCC formation and progression is therefore useful for discovering novel treatment approaches and enhancing the prognosis of HCC patients.

Autophagy, literally meaning self‐eating, is a highly conserved cellular degradation process through which cytoplasmic material, aggregated proteins and organelles are delivered to lysosomes for degradation to ensure the turnover of obsolete cellular components.^8^, ^9^ Three types of autophagy have been reported in mammals: microautophagy, macroautophagy and chaperone‐mediated autophagy.^10^, ^11^ Our research concentrated on macroautophagy, here it referred to autophagy. Autophagy has been implicated in human diseases and physiological processes, including cancer, neurodegeneration and ageing.^12^, ^13^, ^14^ Accumulating evidence has shown that autophagy plays a dual role in the development of cancer.^15^, ^16^, ^17^ However, few studies have reported the effects of autophagy on the development and progression of HCC.^18^ Thus, understanding the molecular mechanisms associated with autophagy in HCC is essential. We used data from The Cancer Genome Atlas (TCGA) and Gene Expression Profiling Interactive Analysis (GEPIA) databases to identify Ubiquitin A‐52 residue ribosomal protein fusion product 1 (UBA52) as one of the most highly expressed autophagy‐related genes in liver cancer tissue. At present, the precise mechanism by which UBA52 modulates autophagy in HCC is unknown.

UBA52, one of the genes associated with ubiquitin, encodes a fusion protein consisting of ubiquitin at the N‐terminus and ribosomal protein L40 at the C‐terminus.^19^, ^20^ Recently, UBA52 has been reported to contribute to the development and progression of various diseases, including colorectal cancer and non‐small cell lung cancer.^21^, ^22^, ^23^ However, few studies have demonstrated that molecular processes through which UBA52 promotes the tumorigenesis and development of HCC. Our goal was to demonstrate how UBA52 affects HCC cell growth and identify novel effective treatment targets for the disease.

In this work, we showed that UBA52 promotes the proliferation and migration of HCC cells, as well as that an elevated level of UBA52 is associated with a poor prognosis in HCC patients. Furthermore, we demonstrated that UBA52 knockdown inhibits HCC cell proliferation and migration through regulating autophagy via EMC6. Moreover, we demonstrated that blocking UBA52 inhibits HCC proliferation and metastasis in vivo. Our research uncovered a brand‐new molecular pathway for the tumorigenesis and development of HCC, and these results suggest that UBA52 may be a new treatment target for patients with HCC.

## 2 MATERIALS AND METHODS

### 2.1 Patient samples and immunohistochemistry (IHC) staining

The First Affiliated Hospital of Anhui Medical University's Department of General Surgery provided samples from individuals with clinically confirmed HCC (*n* = 6). The criteria for confirming HCC include: 1. Imaging studies: ultrasound, computed tomography (CT), magnetic resonance imaging (MRI) or angiography; 2. Alpha‐fetoprotein (AFP) levels; 3. Sample for histopathological examination; 4. Clinical history and risk factors: chronic hepatitis B or C infection, alcohol abuse, non‐alcoholic fatty liver disease, etc. The inclusion criteria HCC patients in the study are as follows: 1. patients diagnosed with HCC clinically and pathologically; 2. patients who have not undergone any other treatment before surgery; 3. patients without other tumours. Table S1 displays the clinicopathological features of the samples. Informed permission was given by every patient. Tumour samples from HCC patients undergoing surgical resection were harvested, and matching the adjacent tissues to cancer were also obtained. Adjacent tissues to cancer were located 1 cm away from the tumour edge. The adjacent tissues to cancer were used for control in the study. After receiving permissions from The First Affiliated Hospital of Anhui Medical University's ethical committee, analysis was carried out. Standard methods were used to fix tumour samples in 4% paraformaldehyde and embed them in paraffin. The primary antibody was incubated with tissue slices on slides for a whole night at 4°C. The primary antibody is described in Table S2. The slides were then washed in phosphate‐buffered saline (PBS), stained with 3,3′‐diaminobenzidine (ZSGB‐BIO, Beijing) and counterstained with haematoxylin before being treated with anti‐rabbit or anti‐mouse IgG (ZSGB‐BIO) as the secondary antibody. The sections were scanned with a tissue scanner (TissueFAXS Plus S, TissueGnostics, Austria; or Pannoramic SCAN, 3DHISTECH, Hungary), and the images were then digitized. The areas of positive immunohistochemical staining in the sections were analysed by ImageJ 1.53 software.

### 2.2 Cell culture

The cell line from the human normal liver (LO2), the cell line from the human embryonic kidney (293T), and four cell lines from human liver cancer (HepG2, Snu449, Huh7 and Hep3B) were bought from the Shanghai Institute of Cell Research, Chinese Academy of Sciences. Short tandem repeat (STR) profiling was used for verifying cell line authentication. HepG2, Snu449, Huh7 and 293T cells were cultured in DMEM (Gibco) supplemented with 10% FBS (Clark Bioscience) and 1% penicillin and streptomycin (Beyotime Biotechnology); LO2 cells were cultured in DMEM supplemented with 20% FBS and 1% penicillin and streptomycin; Hep3B cells were cultured in RPMI‐1640 medium (Gibco) supplemented with 10% FBS and 1% penicillin and streptomycin; and all cells were incubated in a humidified incubator (Thermo).

### 2.3 Bioinformatics analysis

The TCGA (portal.gdc.cancer.gov/projects/TCGA‐LIHC) and GEPIA (gepia2.cancer‐pku.cn/#degenes) databases were used to retrieve the gene expression information for HCC samples.^24^, ^25^ The TCGA‐liver hepatocellular carcinoma (LIHC) database contained a total of 374 HCC patients. Table S1 displays the clinicopathological characteristics of the cohort's patients. The Database for Annotation, Visualization and Integrated Discovery (DAVID) tool (https://david.ncifcrf.gov/conversion.jsp) was used to analyse gene ontology. Using the R program, differential gene expression analysis between the HCC and normal tissues was carried out. Using the clusterProfiler tool, KEGG enrichment analysis was carried out. To indicate statistical significance, a significance threshold of 0.05 and the false discovery rate of 0.05 were used. Using Gene Set Enrichment Analysis (GSEA; http://www.broad.mit.edu/gsea/), further pathway analysis was carried out. The Human Protein Atlas (HPA) database was used to find published data from tissue microarrays of tumour and normal tissues (www.proteinatlas.org/search/UBA52). Using the R survival package, survival analysis was carried out using data from the TCGA and GEPIA databases. The data were analysed with R statistical software (https://www.r‐project.org/).

### 2.4 Western blotting analysis

Using the radioimmunoprecipitation assay (RIPA) lysis method, cells or tissues were collected, and total protein was then extracted. Supernatants from the centrifugation of protein samples were kept at −80°C until they were used in the Western blot analysis. Before being put onto nitrocellulose membranes, samples of protein were broken down using 12.5% or 15% SDS/PAGE. The membranes containing proteins have been treated with specific antibodies for a whole night at 4°C after being blocked with 5% nonfat dry milk for a period of 1.5 h. Prior to imaging, protein‐containing membranes were treated with secondary antibodies and then with a chemiluminescence reagent (Zenbio, Chengdu, China). Finally, the protein band densities were calculated using ImageJ software. The expression levels of the targeted proteins were compared to those of β‐actin to establish a baseline. The antibodies are described in Table S2.

### 2.5 Coimmunoprecipitation (co‐IP)

For each immunoprecipitation procedure, 1 mg of the whole‐cell extract from Huh7 cells that had been lysed with lysis buffer was employed. Immunoprecipitations were performed using the specified antibodies and control rabbit lgG overnight at 4°C. Lysis buffer was used to lyse 293T cells contained FLAG‐UBA52 and HA‐EMC6 plasmids. Following that, the cell lysates were treated overnight with anti‐FLAG and anti‐HA antibodies (Table S2). Protein A/G magnetic beads were used to collect the immune complexes (MCE, Shanghai, China). The immune complexes were then magnetically purified. The immune complexes were collected and analysed using Western blotting after being washed in PBS. Coimmunoprecipitation was repeated three times.

### 2.6 RNA sequencing (RNA‐seq)

Samples were sent to Biomarker Technologies Corporation (Beijing, China) to carry out the RNA‐seq. RNA‐Seq analysis was conducted with three replicates. The TRIzol method was used to extract the total RNA from Huh7 cells with UBA52 knockdown or negative control (NC) Huh7 cells. A NanoDrop 2000 spectrophotometer was used to measure the amount and quality of RNA of Huh7 cells. RNA‐seq libraries were produced in accordance with the manufacturer's instructions. Fold change (FC) ≥ 2 and an adjusted *p* value <0.01 were used as criteria to find differentially expressed genes, which were visualized on a heatmap and a volcano plot. The accession number for the raw RNA‐seq information from Huh7 cells in the Gene Expression Omnibus (GEO) is GSE235017.

### 2.7 Transmission electron microscopy (TEM)

After Huh7 cells of the different groups were fixed with 2.5% glutaraldehyde for 4 h at low temperatures, the cells were then cleaned with PBS and postfixed with 1% OsO4 buffer for 2 h. Samples were cleaned, dehydrated in different concentrations of ethanol solutions and implanted in epoxy resin (Epon812). The samples were subsequently cut into portions (70 nm thick). Uranyl acetate was used to stain the ultrathin slices, and lead citrate was used as a counterstain. The samples were then analysed using a TEM (Thermo, Talos L120C, USA; or JEOL, JEM1400, Japan).

### 2.8 Plasmid construction, short hairpin RNA (shRNA) and small interfering RNA (siRNA) design, transfection/infection

The siRNA targeting UBA52/EMC6 was designed by GenePharma (Shanghai, China). As a control, siNC (a negative control siRNA) was performed. Both UBA52 overexpression plasmid containing a FLAG tag (FLAG‐UBA52) and EMC6 overexpression plasmid containing an HA tag (HA‐EMC6) were purchased from YouBio (Hunan, China). The Lipofectamine 2000 Reagent was transfected in accordance with the manufacturer's instructions (Life Technologies, Carlsbad, USA). At a suitable concentration of 20 nM, the combination of siRNA was added to the HepG2 and Huh7 cells after they had reached 50% density. After 24 or 48 h of culture, the cells were ready for use. 293T cells were treated with recombinant plasmids that expressed Flag‐UBA52 and HA‐EMC6 with Lipofectamine 2000 reagent and collected after 24 h of transfection for the co‐IP experiment. The lentiviral vector carrying the shRNA targeting UBA52, the lentiviral UBA52 overexpression vector and the negative control lentiviral vector were obtained from GenePharma. The polybrene (5 g/L; GenePharma) was used to facilitate the infection of cells with lentiviral particles. The supernatant was replaced with full culture media 24 h after infection. Before being used in further investigations, transduced cells were screened with 2.5 μg/mL puromycin (GenePharma) for 3–5 days after being infected for 72 h. Table S3 provides the complete list of plasmid sequences.

### 2.9 Real‐time quantitative PCR (qRT‐PCR)

Using an RNA extraction Kit (Omega), total RNA of cultured cells and tissues was extracted. Utilizing a cDNA First Strand Synthesis Kit (Bioer), cDNA was obtained. SYBR Green reagents (Bioer) were used to perform qRT‐PCR on a Stratagene Mx3000P qPCR system (Agilent, Santa, USA). Using the comparative 2^−ΔΔCT^ approach, relative gene expression was assessed. The expression levels of the target mRNA were adjusted to those of GAPDH. Primer pairs obtained from Sangon Biotech are listed in Table S4.

### 2.10 Immunofluorescence staining

To visualize GFP‐RFP‐LC3 puncta, HCC cells were infected with eGFP‐mRFP‐LC3 lentivirus, which was purchased from General Biological (Anhui, China). Viral infection was performed as previously described. The targeted siRNA or siNC was introduced to the eGFP‐mRFP‐LC3 lentivirus‐infected HCC cells for a period of 24 h. After treatment, cells were seeded on cover slides (Sail Brand, China), and then they were postfixed in 4% paraformaldehyde for 15 min. Fluorescence microscopy was used to see the cells and take pictures of them (Leica, Wetzlar, Germany).

### 2.11 Cell Counting Kit‐8 (CCK‐8) and colony formation assays

Three thousand cells were sown into each well of 96‐well plates before being overnight cultured in an incubator. HCC cells were then treated with CCK‐8 reagent (Epizyme, Shanghai, China) and incubated for 1–2 h at the appropriate temperature. At 450 nm, optical density (OD) values were determined. At 24, 48 and 72 h, the absorbance of cells in each well was assessed using a microplate reader (Bio‐Tek, US). In six‐well plates, the colony formation test was carried out. In the media containing 10% FBS, cells were planted at a density of 4000 cells per well. For 1–2 weeks, cells treated with various treatments were grown until colonies could be seen clearly. Colonies were preserved with methanol, dyed with 0.1% crystal violet solution and counted using ImageJ software.

### 2.12 Cell migration assay

Following the manufacturer's protocol, the Transwell migration experiment was conducted (BIOFIL, Guangzhou, China). In brief, the lower chamber of the Transwell insert received 500 μL of DMEM containing 10% FBS, while the upper chamber received 1 × 10^5^ cells mixed in serum‐free medium. After being cultivated in an incubator for 24 h, the migrating cells were fixed and dyed. Nonmigrated cells were eliminated from the upper chamber using tools. A digital camera that was attached to an inverted microscope (Leica, Wetzlar, Germany) captured Images of the migratory cells. The migrated cells were quantified by using ImageJ software.

### 2.13 Wound healing assay

The HCC cells on the 6‐well plate had reached 90% confluency after being cultured. With a pipette tip, a wound was made by scratching the cell layer's surface. After washing in PBS, the original medium was cleaned and then replaced with FBS‐free culture medium. A light microscope (Leica, Wetzlar, Germany) was used to take pictures of the injured regions, and wound closure was shown at 0 and 24 h after scratching. The formula used to determine the percentage of wound closure was expressed in the following way: wound closure (%) = [(free cell area at 0 h − free cell area at 24 h)/free cell area at 0 h] × 100%.

### 2.14 Animal experiments

The Committee on the Ethics of Animal Experiments of Anhui Medical University (No. LLSC20221082) gave us permission to conduct all animal experiments in strict accordance with principles. GemPharmatech Co., Ltd. (Jiangsu, China) provided the male nude mice (BALB/c‐nu, age: 4–5 weeks) for experiments. Injecting 5 × 10^6^ infected Huh7 cells (Huh7^LV−shNC^ or Huh7^LV−shUBA52^ cells, respectively) suspended in 200 μL of PBS into the left axillae of nude mice separated into two groups at random. Following a 6‐week observation period, all the nude mice were sacrificed. The subcutaneous tumours were obtained, and the weight and volume were assessed. The subcutaneous tumours were fixed with formalin, paraffin embedded, sectioned and stained following a standard IHC staining protocol. In addition, Injecting 2 × 10^6^ Huh7^LV−shNC^ or 2 × 10^6^ Huh7^LV−shUBA52^ cells mixed in 200 μL of PBS into the tail vein of nude mice separated into two groups at random. All of the mice were sacrificed and their lungs were taken after 6 weeks. haematoxylin and eosin (HE) was used to stain the lung tissue after it had been paraffin embedded. To visualize the expression of relevant molecules, IHC was performed on subcutaneous tumours and lungs.

### 2.15 Statistical analysis

The Kolmogorov–Smirnov test was used to check the normal distribution of the data. Normal distribution variables were presented as mean ± standard deviation (SD), and skewed variables were expressed as median with interquartile range (IQR). Student's *t* and Mann–Whitney *U* tests were used to compare the means of normal and asymmetric variables, respectively. A one‐way analysis of variance (ANOVA), followed by a LSD (Least Significant Difference) test were used to compare three or more groups. Considering the multiple comparisons, Benjamini‐Hochberg (BH) method was applied to regulate the false discovery rate (FDR). *p*‐Value less than 0.05 were considered as statistically significant difference. Software from GraphPad Prism 8.0 and SPSS 22.0 were used for the statistical analysis.

## 3 RESULTS

### 3.1 Increased levels of UBA52 expression correlate with poor prognosis in HCC patients

**FIGURE 1:**
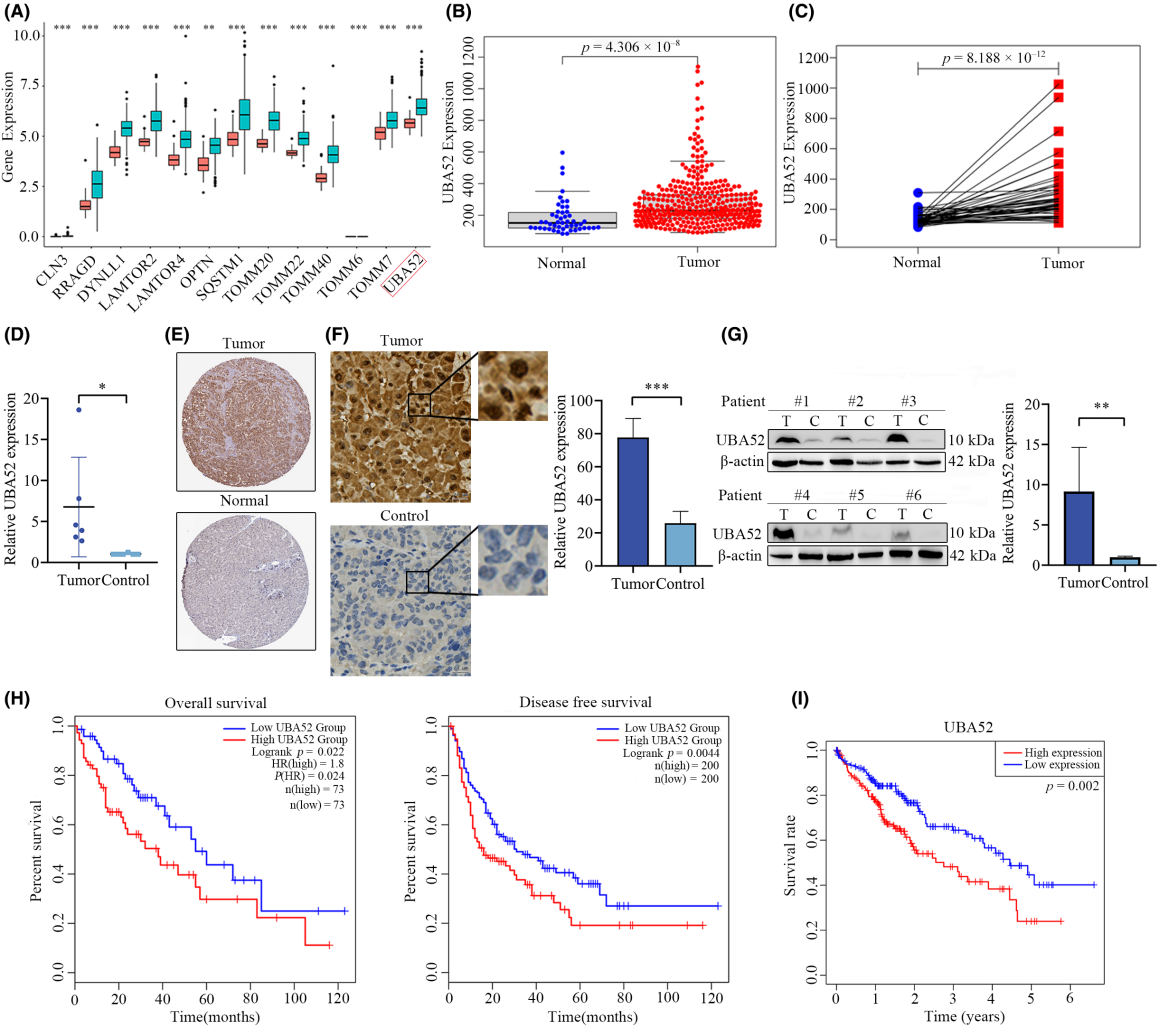
The level of UBA52 is increased in HCC patients and negatively correlated with the prognosis of HCC patients. (A) Subsets of upregulated autophagy‐related genes were identified in the LIHC cohort in the GEPIA database using the DAVID tool. The red boxplot shows gene expression in the normal group, and the blue boxplot shows gene expression in the tumour group. (B) Dot plot showing UBA52 expression in HCC tumour and normal tissues in the TCGA database. (C) Line plot showing UBA52 expression in HCC tumour samples and paired normal tissues in the TCGA database. (D) qRT‐PCR analysis results showing the mRNA level of UBA52 in paired tumour and control liver tissues of HCC patients. (E) Tissue microarray analysis results showing the expression of UBA52 in HCC tumour and normal liver tissues in the HPA database. (F) UBA52 expression was detected by IHC staining in paired tumour and control tissues of HCC patients. (G) Western blot analysis results showing the protein expression of UBA52 in paired tumour and control tissues of HCC patients. (H) Kaplan–Meier survival curve showing that UBA52 expression correlates with OS (left graph) and DFS (right graph) in HCC patients in the GEPIA database. (I) Kaplan–Meier survival curve showing the correlation of UBA52 expression with the survival rate of HCC patients in the TCGA database. The patients were divided into the high and low expression groups based on the median expression level of UBA52. *p < 0.05, **p < 0.01, ***p < 0.001. T, tumour; C, control tissues; HR, hazard ratio.

From the LIHC cohort in the GEPIA database, 1482 upregulated differentially expressed genes were chosen. Using the DAVID online tool, we further screened for autophagy‐related genes and discovered that UBA52 was one of the most highly expressed autophagy‐related genes (Figure 1A). Additionally, we discovered that UBA52 mRNA expression level in LIHC tissues was significantly higher than it was in the normal liver tissues from the TCGA database (Figure 1B,C). Furthermore, we verified these results using qRT‐PCR on samples from our cohort (Figure 1D). The UBA52 protein expression level was higher in tumour tissues in HPA database by tissue microarray analysis (Figure 1E). Using IHC staining and western blot, we then validated these results in our cohort (Figure 1F,G). Moreover, in the GEPIA database, patients with high levels of UBA52 had significantly poorer survival outcome than patients with low levels of UBA52 (Figure 1H). Similar to this, elevated levels of UBA52 in the TCGA database for HCC patients were linked to a poor prognosis (Figure 1I). These findings showed the UBA52 expression level was negatively correlated with patient prognosis.

### 3.2 UBA52 knockdown suppresses the proliferation and migration of HCC cells

**FIGURE 2:**
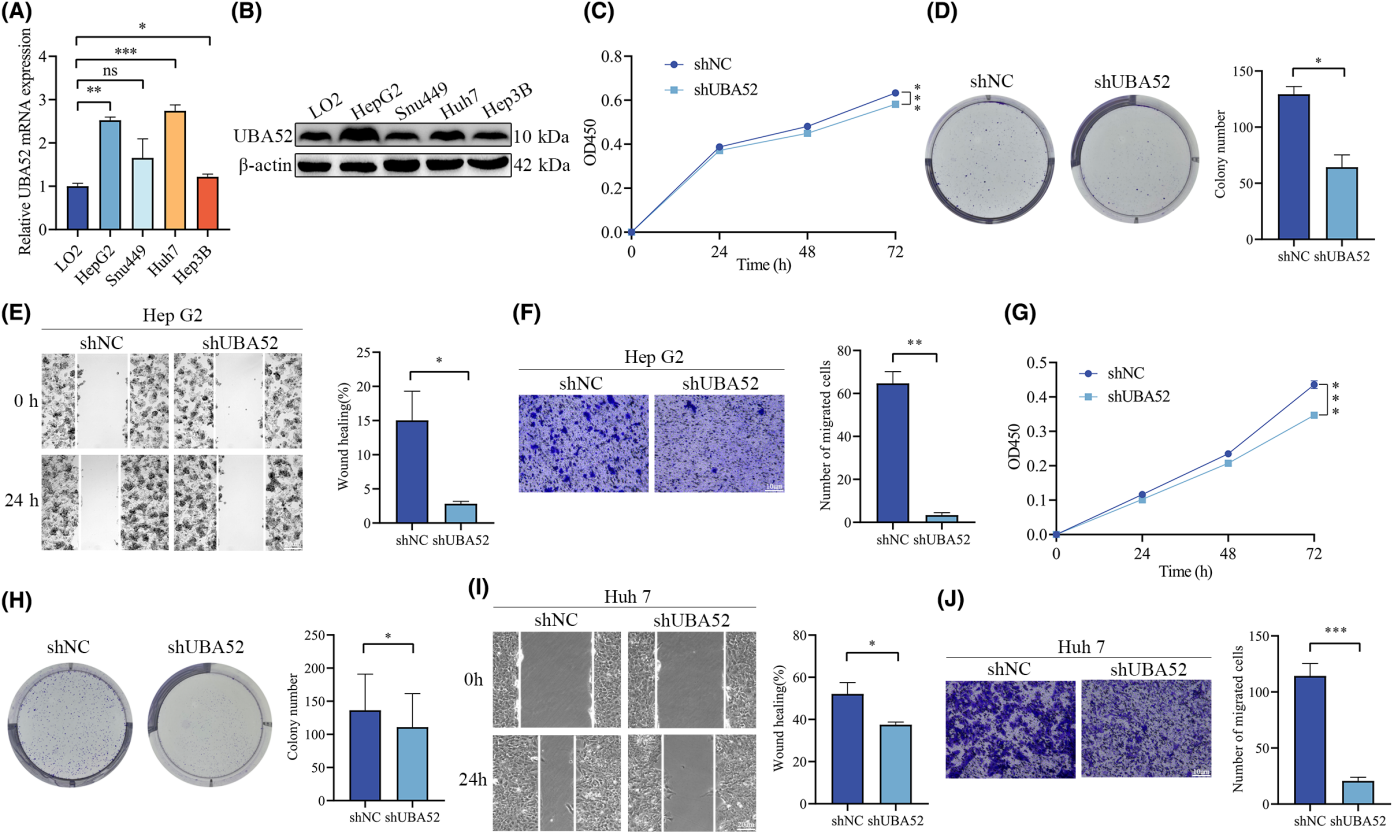
Knockdown of UBA52 suppresses the proliferation and migration of HCC cells. (A) qRT‐PCR analysis results showing the mRNA level of UBA52 in four HCC cell lines with different migration and invasion abilities and a normal liver cell line. (B) Western blot analysis results showing the protein level of UBA52 in four HCC cell lines with different migration and invasion abilities and a normal liver cell line. (C, G) A CCK‐8 assay was used to evaluate the proliferation ability of HepG2 (C) and Huh7 (G) cells in the shNC group and shUBA52 group. (D, H) A colony formation assay was used to evaluate the proliferation ability of HepG2 (D) and Huh7 (H) cells in the shNC group and shUBA52 group. The bar graph on the right shows the quantification of clonogenicity (*n* = 3, mean ± SD). (E, I) A wound healing assay was performed to evaluate the migration ability of HepG2 (E) and Huh7 (I) cells in the shNC group and shUBA52 group. Scale bar = 20 μm. (F, J) A Transwell assay was performed to evaluate the migration ability of HepG2 (F) and Huh7 (J) cells in each group. Scale bar = 10 μm. *p < 0.05, **p < 0.01, ***p < 0.001. OD450, optical density at 450 nm; shNC, negative control short hairpin RNA; shUBA52, short hairpin RNA targeting UBA52.

We checked the UBA52 expression levels in four HCC cell lines with different proliferation and migration abilities, including HepG2, Snu449, Huh7 and Hep3B, as well as in the liver cell line LO2, in order to investigate the impact of UBA52 on the HCC cells proliferation and migration. For further study, we selected the HepG2 and Huh7 with the highest levels of UBA52 (Figure 2A,B). Lentivirus was used to infect HepG2 and Huh7 cells to silence UBA52 expression. ShUBA52#3 and shUBA52#4, which induced more significant knockdown effect, were selected for knockdown of UBA52 in HepG2 and Huh7 cells, respectively (Figure S1A,B). Considering that UBA52 might impact the proliferation of cells,^26^ we examined the effect of UBA52 knockdown on HCC cell proliferation and migration. Next, we carried out CCK‐8 and colony formation experiments and discovered that UBA52 knockdown significantly decreased the proliferation capacity of HepG2 cells (Figure 2C,D). Furthermore, we performed Transwell and wound healing experiments and found that UBA52 knockdown significantly decreased the migration capacity of HepG2 cells (Figure 2E,F). Similarly, the Huh7 cells proliferation and migration capacities were decreased significantly in the shUBA52 group (Figure 2G–J). In addition, we chose the Hep3B cell line, which expresses UBA52 at the lowest level, to investigate the impact of UBA52 overexpression on HCC cells (Figure 2A,B). Hep3B cells were infected with LV‐UBA52 lentivirus to overexpress UBA52 (Figure S2A,B). We found that similar results following UBA52 overexpression in Hep3B (Figure S2C–F). Thus, our results demonstrated that UBA52 knockdown suppressed the HCC cells proliferation and migration.

### 3.3 Knockdown of UBA52 promotes autophagic flux in HCC cells

**FIGURE 3:**
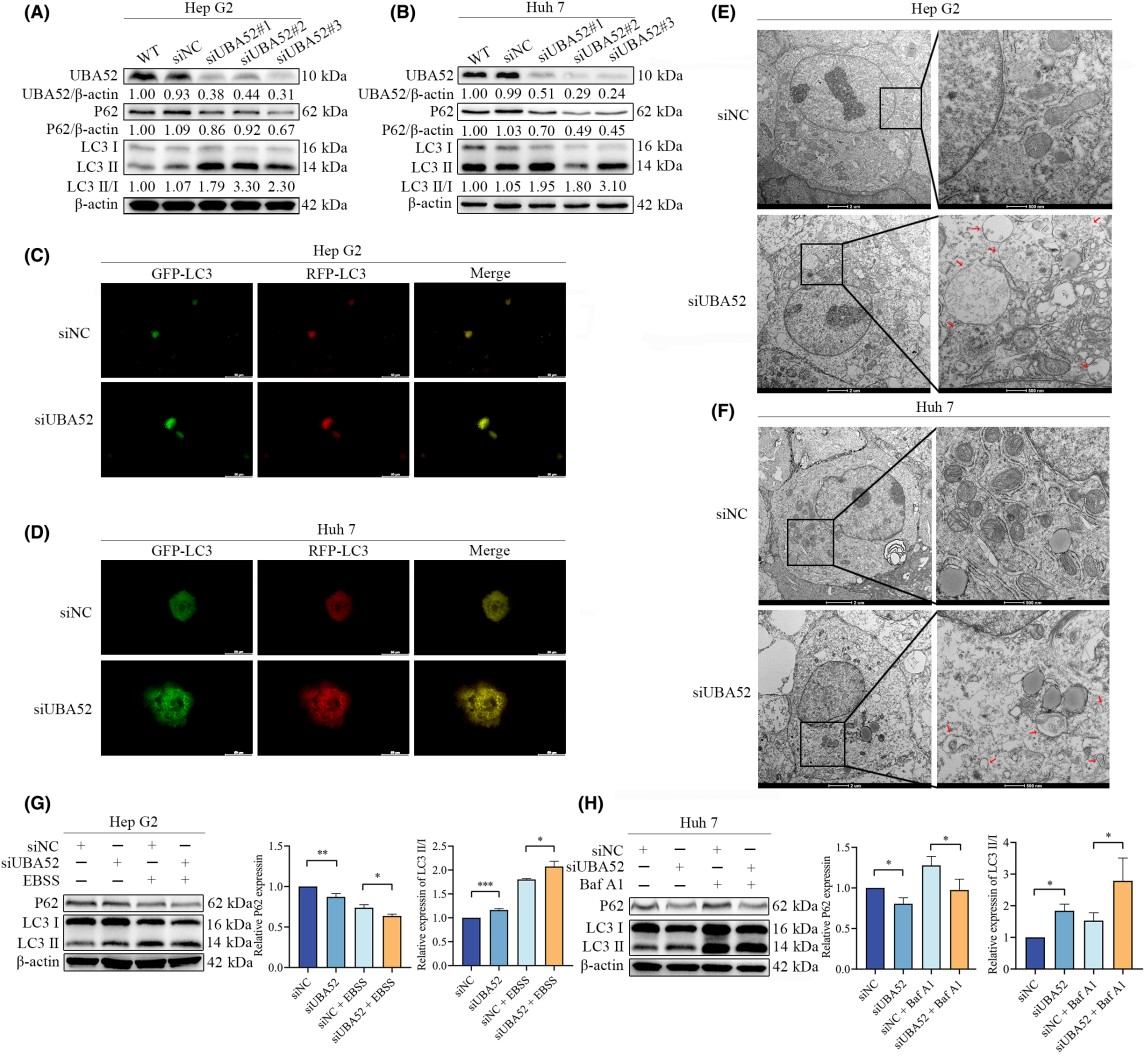
Knockdown of UBA52 promotes autophagic flux in HCC cells. (A, B) Western blot analysis results showing UBA52, P62, LC3I and LC3II protein expression in HepG2 (A) and Huh7 (B) cells. (C, D) The rate of autophagic flux was determined by using fluorescence microscopy in HepG2 (C) and Huh7 (D) cells infected with eGFP‐mRFP‐LC3 lentivirus. Scale bar = 50 μm. (E, F) Electron micrographs showing autolysosomes in HepG2 (E) and Huh7 (F) cells transfected with siUBA52 and siNC, respectively. The red arrowhead indicates autolysosomes. Scale bar = 0.5–2 μm. (G) Western blot analysis results showing the protein levels of P62, LC3I and LC3II in HepG2 cells transfected with siUBA52 or siNC and treated with or without EBSS for 6 h. (H) Western blot analysis results showing the protein levels of P62, LC3I and LC3II in Huh7 cells transfected with siUBA52 or siNC and treated with or without Baf A1 (100 nM). *p < 0.05, **p < 0.01, ***p < 0.001. WT, wild type; siNC, negative control small interfering RNA; siUBA52, small interfering RNA against UBA52; EBSS, Earle's balanced salt solution; Baf A1, bafilomycin A1.

We further investigated the connection between UBA52 and autophagy in HCC cells because our earlier findings showed that UBA52 was tightly connected with autophagy in the GEPIA database. Three different siRNAs (siUBA52#1, #2 and #3) specifically targeting UBA52 were transfected into HepG2 and Huh7 cells to silence UBA52 expression. SiUBA52#3, which induced the most significant knockdown, was selected for knockdown of UBA52 expression in these two types of cells (Figure 3A,B). First, we examined the association between UBA52 and autophagy‐related genes in HCC cells. The amount of P62 decreased and the ratio of LC3II to LC3I rose when UBA52 expression was knocked down (Figure 3A,B). Additionally, using fluorescence microscopy, we discovered that knockdown of UBA52 had more LC3 puncta in HepG2 and Huh7 cells (Figure 3C,D). Knockdown of UBA52 also led to an increase in intracellular autolysosomes (Figure 3E,F). Moreover, we cultured HepG2 cells with EBSS, which is used as a positive control reagent to induce autophagy, for starvation treatment. Comparing the EBSS and UBA52 knockdown groups to the group acting as a control, the expression level of P62 decreased and the ratio of LC3II to LC3I rose; especially, the level of autophagy was highest in the EBSS + siUBA52 group (Figure 3G). Moreover, we treated Huh7 cells with Baf A1 (an autophagy inhibitor) for an autophagic flux assay and discovered that the ratio of LC3II to LC3I further rose (Figure 3H). In total, our findings showed that knockdown of UBA52 increased autophagic flux in HCC cells.

### 3.4 Knockdown of UBA52 increases EMC6 expression

**FIGURE 4:**
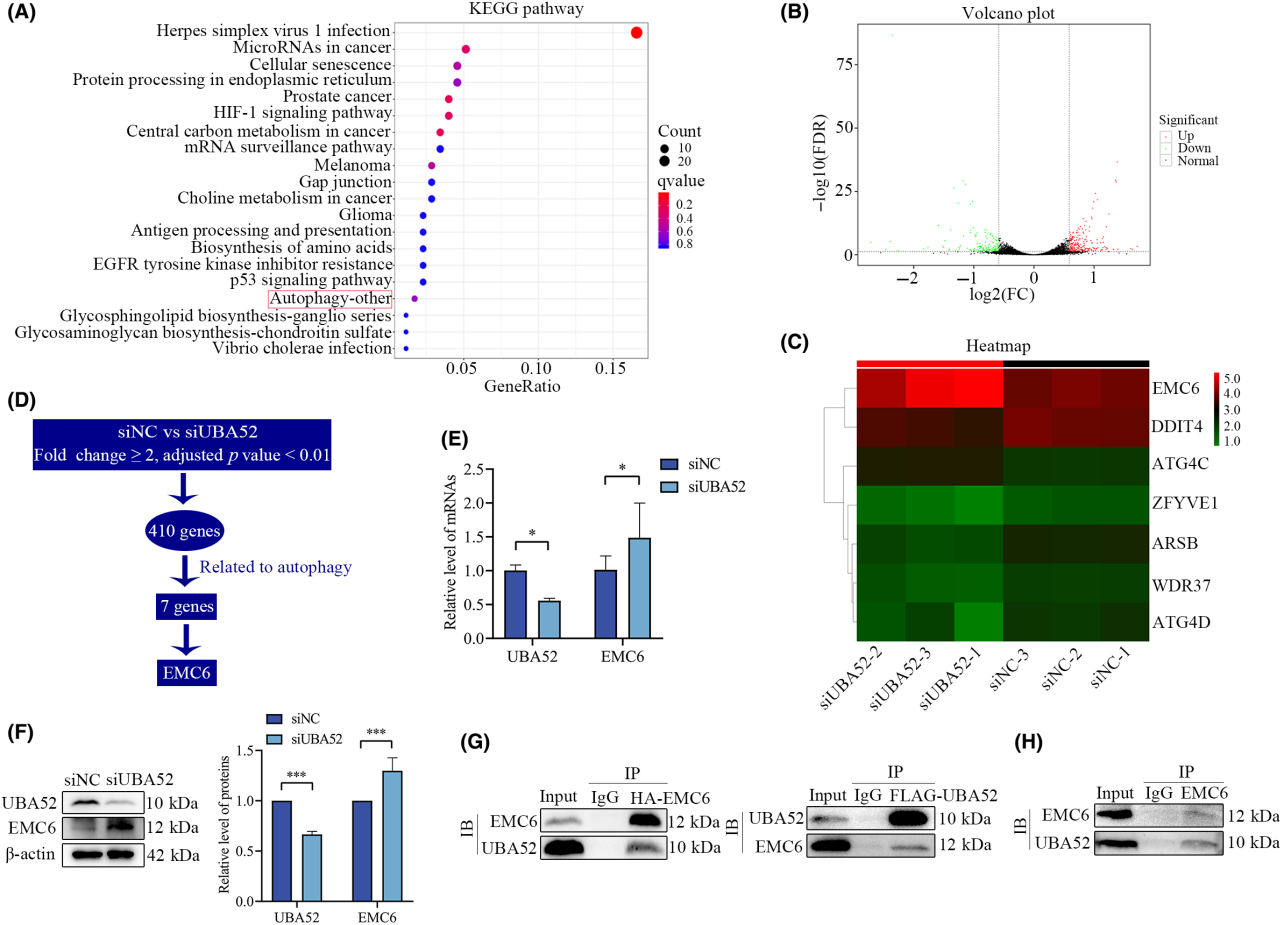
Knockdown of UBA52 increases EMC6 expression. (A) KEGG pathway enrichment analysis results showing the top 20 pathways enriched with differentially expressed genes between the siNC group and siUBA52 group. (B) Volcano plot showing differential gene expression in the siNC group and siUBA52 group. (C) Heatmap showing the differentially expressed autophagy‐related genes in the siNC group and siUBA52 group. (D) Flowchart showing the process used to screen for the potential downstream molecules. (E) qRT‐PCR analysis results showing the mRNA level of EMC6 in Huh7 cells with UBA52 knockdown. (F) Western blot analysis results showing the protein level of EMC6 in Huh7 cells with UBA52 knockdown. (G) Co‐IP assay with FLAG‐UBA52 and HA‐EMC6 showing the interaction between UBA52 and EMC6 in 293T cells. (H) Endogenous IP assay showing that UBA52 can be precipitated by EMC6 in Huh7 cells. *p < 0.05, ***p < 0.001. FDR, false discovery rate; FC, fold change; siNC, negative control small interfering RNA; siUBA52, small interfering RNA against UBA52.

We analysed alterations in gene expression by RNA‐seq analysis to identify the molecular changes induced by UBA52‐mediated regulation of autophagy in HCC cells. The biological data between the NC group and the UBA52 knockdown group was compared, and KEGG enrichment analysis revealed that UBA52 was closely associated with autophagy pathways (Figure 4A). We discovered 410 differentially expressed genes using volcano plot analysis: 250 upregulated genes and 160 downregulated genes (Figure 4B). By screening these genes, we discovered seven autophagy‐related genes (Table S5), and EMC6 was one of the genes with the most noticeably altered levels of expression in this category (Figure 4C,D). Validation analyses showed that UBA52 knockdown increased EMC6 expression (Figure 4E,F). To verify whether UBA52 interacts with EMC6, we performed a co‐IP experiment and showed that exogenous UBA52 associates with EMC6 in 293T cells (Figure 4G). Furthermore, UBA52 was precipitated by EMC6 in Huh7 cells according to the endogenous co‐IP experiment results (Figure 4H). These findings indicated that UBA52 knockdown increased the expression of EMC6.

### 3.5 Knockdown of UBA52 suppresses the proliferation and migration of HCC cells by regulating autophagy through EMC6

**FIGURE 5:**
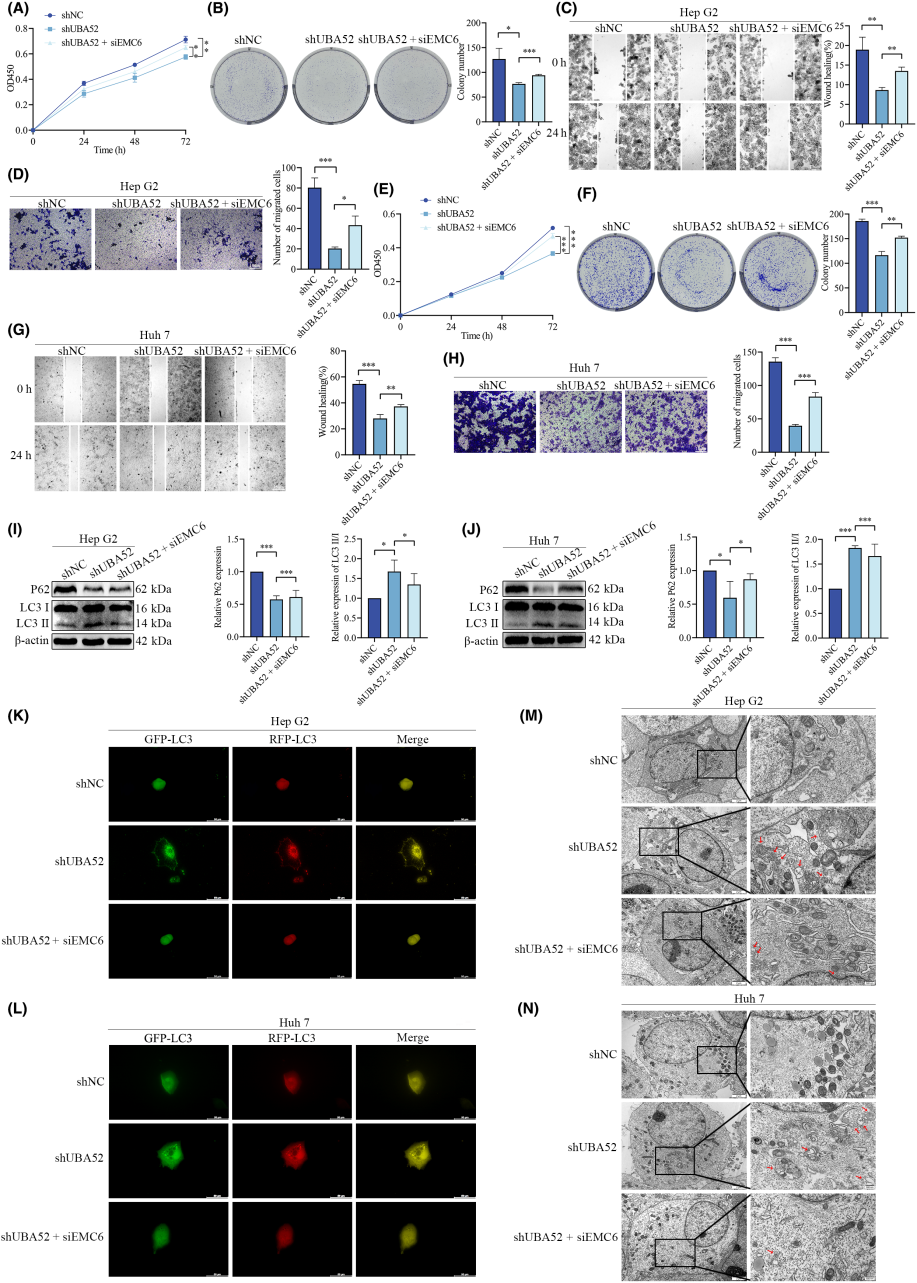
Knockdown of UBA52 suppresses the proliferation and migration of HCC cells by regulating autophagy through EMC6. (A, E) A CCK‐8 assay was used to evaluate the proliferation ability of HepG2 (A) and Huh7 (E) cells in the shNC group, shUBA52 group and shUBA52 + siEMC6 group. (B, F) A colony formation assay was used to evaluate the proliferation ability of HepG2 (B) and Huh7 (F) cells in the shNC group, shUBA52 group and shUBA52 + siEMC6 group. The bar graph on the right shows the quantification of clonogenicity (*n* = 3, mean ± SD). (C, G) A wound healing assay was performed to evaluate the migration ability of HepG2 (C) and Huh7 (G) cells in the shNC group, shUBA52 group and shUBA52 + siEMC6 group. Scale bar = 25 μm. (D, H) A Transwell assay was performed to evaluate the migration ability of HepG2 (D) and Huh7 (H) cells in each group. Scale bar = 10 μm. (I, J) Western blot analysis results showing the protein levels of P62, LC3I and LC3II in HepG2 (I) and Huh7 (J) cells in the shNC, shUBA52 and shUBA52 + siEMC6 groups. (K, L) The rate of autophagic flux was determined by using fluorescence microscopy in HepG2 (K) and Huh7 (L) cells infected with eGFP‐mRFP‐LC3 lentivirus. Scale bar = 50 μm. (M, N) Electron micrographs showing autolysosomes in HepG2 (M) and Huh7 (N) cells in the different groups. The red arrowhead indicates autolysosomes. Scale bar = 0.5–2 μm. *p < 0.05, **p < 0.01, ***p < 0.001. OD450, optical density at 450 nm values; shUBA52, short hairpin RNA targeting UBA52; siEMC6, small interfering RNA against EMC6.

Previous studies have shown that EMC6 positively regulates autophagy and inhibits the migration and invasion of cancer cells.^27^, ^28^, ^29^ To explore the function of EMC6 in liver cancer, we further conducted KEGG pathway analysis in the TCGA‐LIHC cohort and found that EMC6 was related to the autophagy pathway (Table S6). Furthermore, by GSEA analysis, we found that EMC6 expression was positively correlated with the autophagy pathway in HCC (Figure S3A). We used Western blotting to analyse the association between EMC6 and autophagy‐related genes in order to confirm the GSEA results. We first transfected HCC cells with three unique siRNAs (siEMC6#1, #2 and #3). SiEMC6#3, which induced the most significant knockdown, was selected for knockdown of EMC6 expression in HepG2 and Huh7 cells (Figure S3B,C). P62 expression level rose after EMC6 knockdown, but the ratio of LCII to LC3I reduced (Figure S3D,E). These results indicated that knockdown of EMC6 attenuated autophagy, consistent with the GSEA results. We further carried out CCK‐8 and colony formation experiments to confirm whether UBA52 promotes HCC tumorigenesis and progression via EMC6. We discovered that downregulating EMC6 reversed the suppression of cell proliferation induced by UBA52 knockdown in HepG2 cells (Figure 5A,B). Additionally, we carried out Transwell and wound healing experiments, and we discovered that downregulating EMC6 reversed the inhibition of cell migration brought on by UBA52 knockdown in HepG2 cells (Figure 5C,D). Huh7 cells showed comparable outcomes (Figure 5E–H). To explore whether knockdown of UBA52 induces autophagy via EMC6, we measured autophagic flux in the three treatment groups (shNC group, shUBA52 group and shUBA52 + siEMC6 group) of HepG2 and Huh7 cells. In the shUBA52 + siEMC6 group compared to the shUBA52 group, P62 expression level was higher but the ratio of LC3II to LC3I was lower (Figure 5I,J). By using fluorescence microscopy, we also discovered that there were less LC3 puncta in the shUBA52 + siEMC6 group than in the shUBA52 group (Figure 5K,L). In addition, the shUBA52 + siEMC6 group had less autolysosomes (Figure 5M,N). Thus, these results demonstrated that downregulation of EMC6 reduced autolysosome formation brought on by UBA52 knockdown in HCC cells. Overall, our findings demonstrated that UBA52 knockdown suppressed HCC cells proliferation and migration through regulating autophagy via EMC6.

### 3.6 Knockdown of UBA52 inhibits HCC cell proliferation and metastasis in vivo

**FIGURE 6:**
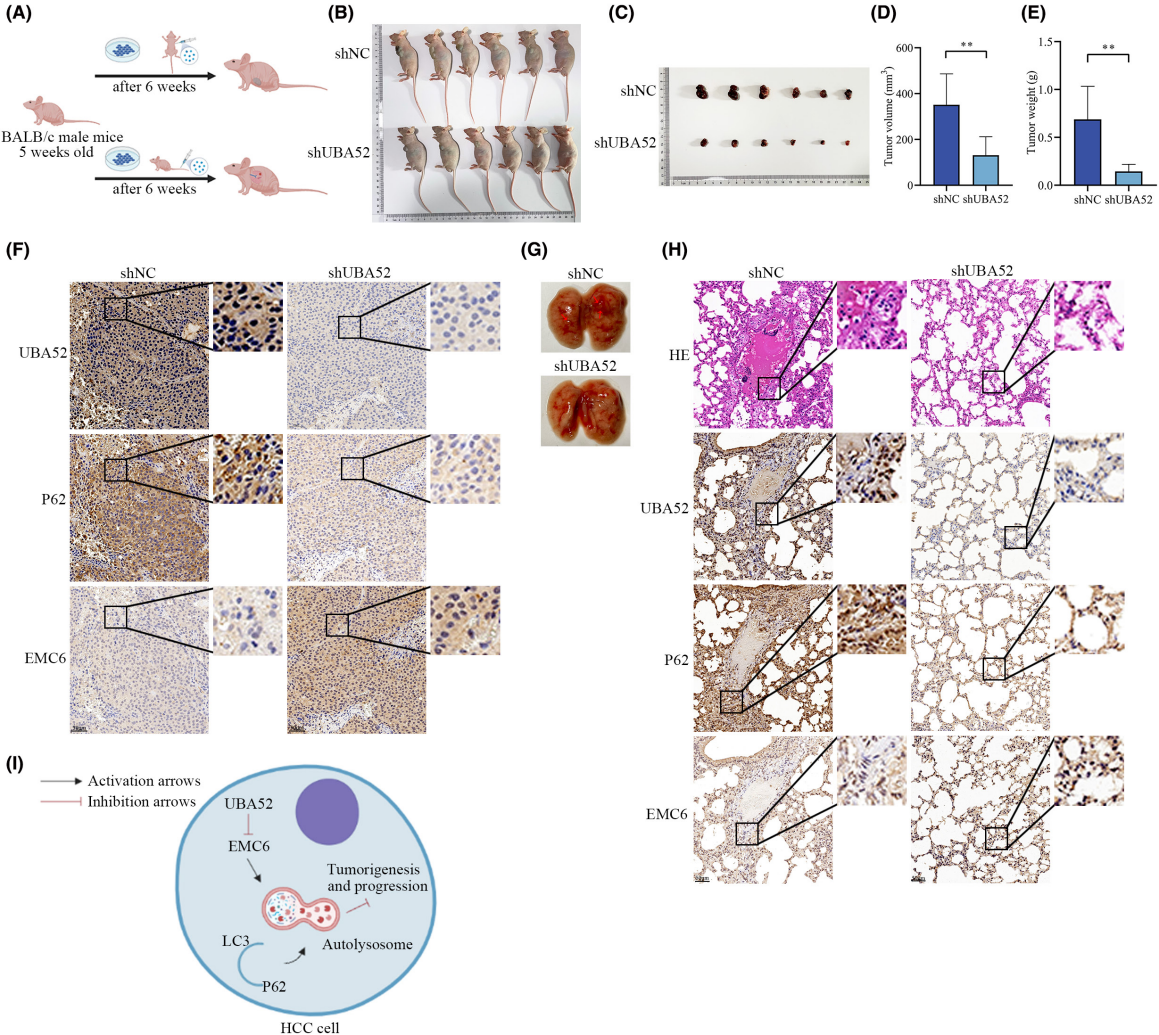
UBA52 knockdown inhibits the proliferation and migration of HCC cells in vivo. (A) Flow diagram showing the experimental procedures for animal studies. (B) Image of tumour‐bearing nude mice in the shNC group and shUBA52 group (*n* = 6 mice per group). (C) Image of subcutaneous xenograft tumours in nude mice in the two groups. The bar graphs show the tumour volume (D) and weight (E). (F) Images of IHC staining of UBA52, P62 and EMC6 in subcutaneous xenograft tumours in the two groups. (G) Image of lung tissues from mice in the shNC and shUBA52 groups in the lung metastasis model. The red arrow indicates a metastatic tumour nodule. (H) Images of HE staining and IHC staining of UBA52, P62 and EMC6 in the lungs of the two groups. (I) A schematic diagram of the mechanism by which UBA52 facilitates HCC tumorigenesis and progression by regulating autophagy through EMC6. **p < 0.01. shNC, negative control short hairpin RNA; shUBA52, short hairpin RNA targeting UBA52; HE, haematoxylin and eosin.

We subsequently established a subcutaneous tumour model and a lung metastasis model in BALB/c nude mice using Huh7 cells to assess the effects of UBA52 in vivo. According to the flowchart of the experimental procedure (Figure 6A), BALB/c nude mice were injected with Huh7 cells subjected to different treatments through the left axilla and tail vein. In comparison to nude mice injected with Huh7^LV−shNC^ cells, Huh7^LV−shUBA52^ cells resulted in subcutaneous tumours that were smaller and lighter (Figure 6B–E). Importantly, the expression level of EMC6 was higher and that of UBA52 and P62 were lower in shUBA52 group compared with shNC group by IHC staining (Figure 6F). In addition, knockdown of UBA52 induced the formation of barely visible metastatic tumours in the lungs (Figure 6G). Comparing the shNC group to the shUBA52 group, HE staining showed obvious tumour metastasis to the lungs (Figure 6H). Moreover, Comparing the shUBA52 group to the shNC group, IHC staining revealed that the expression level of EMC6 was enhanced and that of UBA52 and P62 was reduced (Figure 6H). Our findings collectively showed that UBA52 knockdown suppressed HCC cell proliferation and metastasis in vivo.

## 4 DISCUSSION

Recently, accumulating studies have suggested that autophagy is involved in the development and progression of HCC.^30^, ^31^, ^32^ In our study, we first identified the UBA52 gene, one of the most highly expressed autophagy‐related genes in HCC in the GEPIA database. Then, we collected tissue samples from HCC patients and verified that the UBA52 expression was significantly increased in HCC samples. Moreover, the GEPIA and TCGA datasets showed a correlation between a high UBA52 expression level and a bad outcome in HCC patients. In addition, we revealed a novel molecular mechanism by which UBA52 regulates autophagy via EMC6, promoting the growth and development of HCC.

Autophagy is considered to function as a double‐edged sword in tumour development and plays an essential role in diverse cellular processes.^33^, ^34^ Autophagy plays a tumour suppressor role at the early stage of tumour progression but promotes tumour survival in the late stage.^35^ Our study showed that knockdown of UBA52 induced autophagy in HCC cells, resulting in suppression of cell proliferation and migration. To date, various signalling pathways have been reported to be involved in regulating autophagy during the growth and development of tumours.^36^, ^37^ Zhang et al. found that stimulating autophagy can inhibit liver tumorigenesis via overexpression of DDX5.^38^ In addition, other studies found that SOCS5 inhibition induced autophagy to impair metastasis in HCC.^39^ Consistent with previous research, we found that activation of autophagy led to inhibition of HCC cell growth and migration through UBA52 knockdown. Autophagy‐targeting drugs have become a hotspot in cancer research.^40^ Some studies have found that autophagy‐enhancing drugs can improve the clinical response in patients with HCC. Yu et al. revealed an anti‐HCC mechanism of icaritin via enhancement of autophagy.^41^ Our results suggested that UBA52 could be an autophagy‐related target for the therapy of HCC.

The UBA52 gene encodes ribosomal protein L40, which is associated with extraribosomal functions, including cell proliferation, cell migration and other processes.^42^ Interestingly, we also found that HCC cells proliferate and migrate capacities were reduced by UBA52 knockdown. Although previous studies demonstrated that UBA52 plays a role in Parkinson's disease and embryonic development, few studies have reported the relationship between UBA52 and HCC.^43^, ^44^ In 1995, Barnard et al. discovered that the mRNA level of UBA52 is higher in human colon cancer tissue than in adjacent grossly normal tissue.^45^ Similarly, we confirmed that the expression level of UBA52 is considerably higher in HCC than in liver tissues. Furthermore, our research demonstrated that UBA52 is critical for facilitating HCC tumorigenesis and development both in vitro and in vivo. Considering our results collectively, we reasoned that UBA52 can function as a driver oncogene, eventually leading to HCC tumorigenesis and development.

Through transcriptome sequencing and experimental validation, we found that EMC6 is a downstream molecule regulated by UBA52. EMC6 is an important autophagy‐related molecule that was first discovered by Li et al.^29^ Previous studies have reported that EMC6 suppresses glioblastoma cell proliferation by inducing autophagy.^46^ In our study, we found that downregulation of EMC6 reversed the decreases in HCC cells proliferative and migratory capacities induced by UBA52 knockdown. Furthermore, our findings demonstrated that EMC6 knockdown decreases autophagy level in HCC cells by reducing the conversion of LC3I to LC3II and increasing P62 accumulation. Recently, there have been few reports on the relationship between UBA52 and EMC6. Our study indicated that UBA52 could decrease the expression of EMC6 to promote HCC tumorigenesis and development. In the previous study, we confirmed that downregulating UBA52 expression inhibited the proliferation and migration of HCC cells. To further validate the role of UBA52 in vivo, we injected human HCC cells with suppressed UBA52 expression, establishing xenograft and lung metastasis models in nude mice. Previous studies have reported that in vivo, UBA52 is required for mouse embryo development, which regulates protein synthesis.^47^ However, there is little study investigating the role of UBA52 in the growth of HCC. In our study, we found that downregulating UBA52 expression prevented tumour development and metastasis in mice, which is consistent with our in vitro results. Additionally, we discovered that UBA52 knockdown increased EMC6 expression and decreased P62 expression in mice. These findings may indicate that UBA52 knockdown activates autophagy via EMC6 in vivo.

In summary, our current results reveal a novel UBA52/EMC6/autophagy axis in the regulation of HCC tumorigenesis and progression, as shown in the schematic diagram in Figure 6I. Clinically, a worse outcome was linked to increased UBA52 expression in HCC patients. Mechanistically, UBA52 promoted tumour tumorigenesis and metastasis by inhibiting autophagy and decreasing the expression of EMC6. Our results not only demonstrate a novel molecular mechanism underlying HCC tumorigenesis and progression but also provide insights into the identity of UBA52 as a potential candidate target for the prevention and treatment of HCC.

## AUTHOR CONTRIBUTIONS

**Li Tong:** Conceptualization (equal); writing – original draft (equal). **Xiaofei Zheng:** Formal analysis (equal); project administration (equal). **Tianqi Wang:** Formal analysis (equal); project administration (equal). **Wang Gu:** Formal analysis (equal); project administration (equal). **Tingting Shen:** Data curation (equal). **Wenkang Yuan:** Resources (equal). **Siyu Wang:** Resources (equal). **Songlin Xing:** Data curation (equal). **Xiaoying Liu:** Methodology (equal). **Chong Zhang:** Conceptualization (equal); writing – original draft (equal). **Chao Zhang:** Supervision (equal); writing – review and editing (equal).

## FUNDING INFORMATION

The present study was supported by the University Research Project Foundation of Anhui Province (grant no. 2022AH040161) and Anhui Medical University General Surgery and Cell Biology Joint Project Foundation (grant no. 9001001823).

## CONFLICT OF INTEREST STATEMENT

The authors declare that they have no competing interests.

## Supporting information


Figure S1.

Figure S2.

Figure S3.

Table S1.

Table S2.

Table S3.

Table S4.

Table S5.

Table S6.

Data S1.

## Data Availability

The datasets analyzed during the study are available from the corresponding author upon reasonable request.
