# Supplementary figures and images for: Inhibition of UBA52 induces autophagy via EMC6 to suppress hepatocellular carcinoma tumorigenesis and progression
Source: J Cell Mol Med. 2024 Mar 6;28(6):e18164. doi: 10.1111/jcmm.18164 (PMC10915828; doi:10.1111/jcmm.18164)

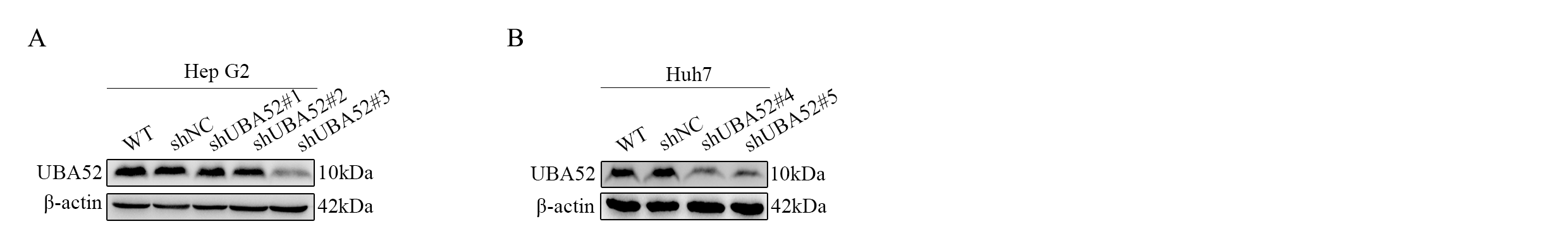

Supplement: Supplementary file 1 — Figure S1. [file JCMM-28-e18164-s008.tif]

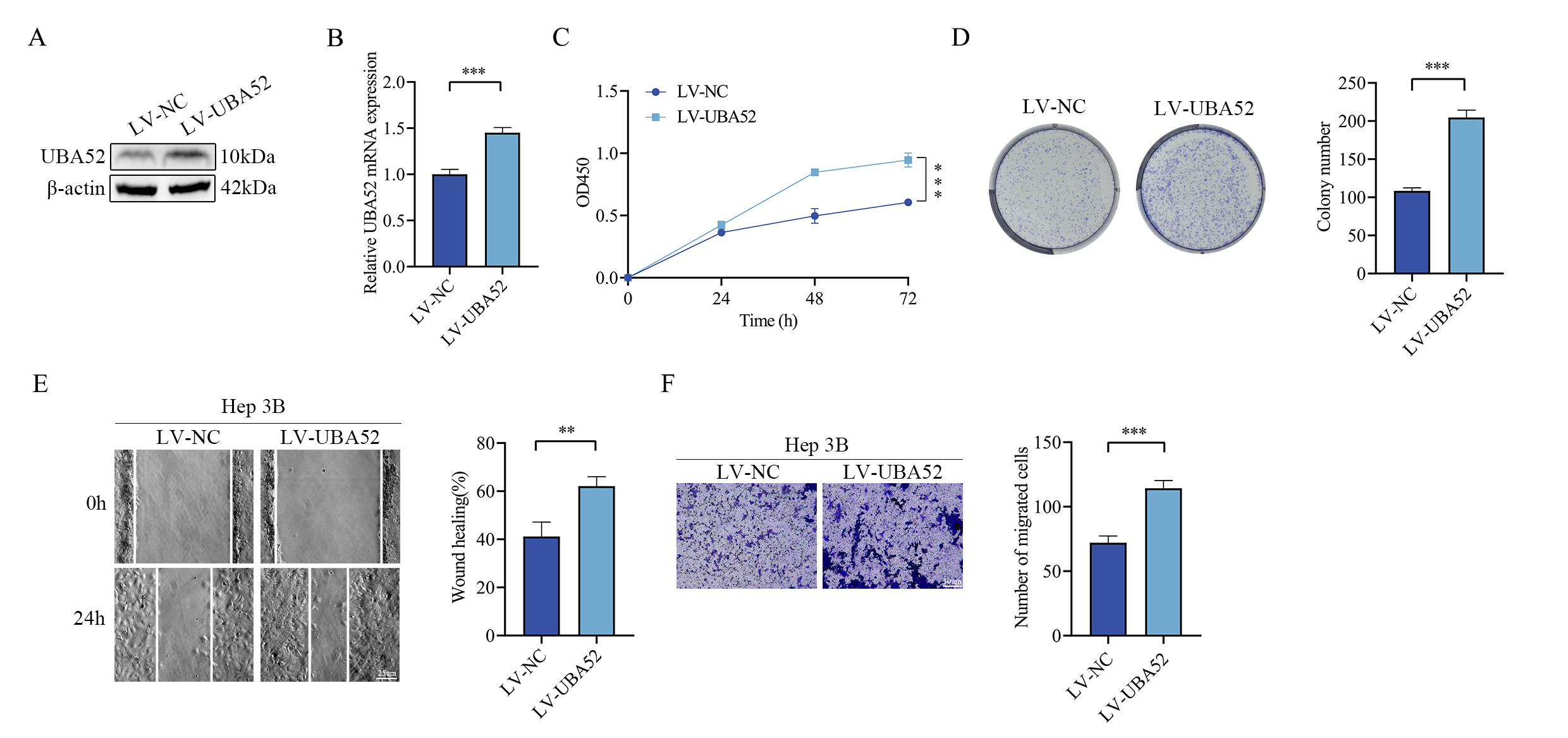

Supplement: Supplementary file 2 — Figure S2. [file JCMM-28-e18164-s004.tif]

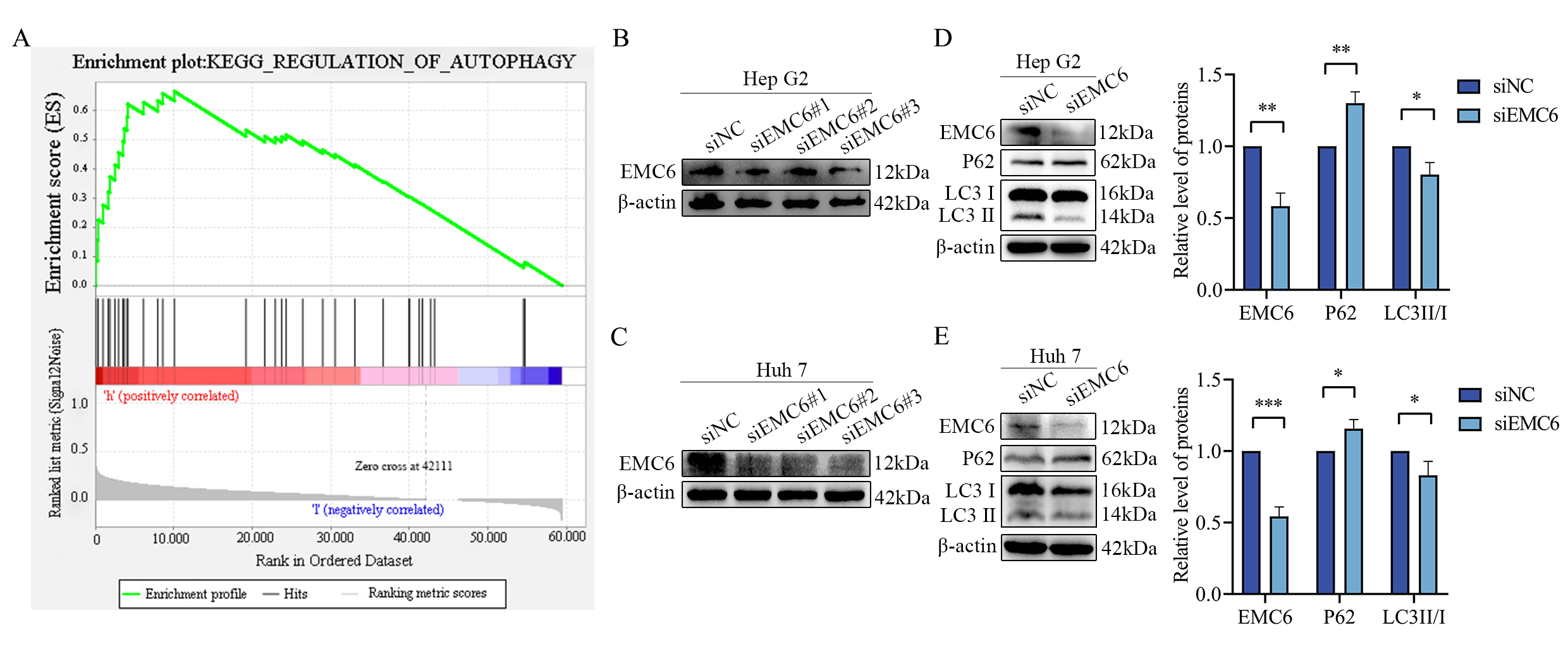

Supplement: Supplementary file 3 — Figure S3. [file JCMM-28-e18164-s009.tif]
