# Supplementary material for: Inhibition of UBA52 induces autophagy via EMC6 to suppress hepatocellular carcinoma tumorigenesis and progression
Source: J Cell Mol Med. 2024 Mar 6;28(6):e18164. doi: 10.1111/jcmm.18164 (PMC10915828; doi:10.1111/jcmm.18164)
Supplement: Supplementary file 4 — Table S1. [file JCMM-28-e18164-s005.doc]

Table SⅠ. Clinicopathologic characteristics of patients in TCGA-LIHC cohort and our patients cohort.

| TCGA-LIHC cohort | | Our patients cohort | |
| --- | --- | --- | --- |
| Variables | Number of samples | Variables | Number of samples |
| Gender |  | Gender |  |
| Male/Female | 253/121 | Male/Female | 4/2 |
| Age |  | Age |  |
| ≤65/>65 | 235/139 | ≤65/>65 | 5/1 |
| Grade |  | Grade |  |
| G1/G2/G3/G4/Unknown | 55/180/123/13/3 | G1/G2/G3/G4/Unknown | 1/4/0/0/1 |
| Stage |  | Stage |  |
| I/II/III/IV/Unknown | 175/86/86/5/22 | I/II/III/IV/Unknown | 3/2/1/0/0 |
| T |  | T |  |
| T1/T2/T3/T4/Unknown | 185/94/81/13/1 | T1/T2/T3/T4/Unknown | 3/2/0/1/0 |
| M |  | M |  |
| M0/M1/Unknown | 271/4/99 | M0/M1/Unknown | 6/0/0 |
| N |  | N |  |
| N0/N1/Unknown | 257/4/113/0 | N0/N1/N2/Unknown | 5/0/1/0 |

TCGA, The Cancer Genome Atlas; LIHC, Liver hepatocellular carcinoma; T, Tumor size and depth of infiltration; M, Metastasis; N, Node.
