# Supplementary material for: Inhibition of UBA52 induces autophagy via EMC6 to suppress hepatocellular carcinoma tumorigenesis and progression
Source: J Cell Mol Med. 2024 Mar 6;28(6):e18164. doi: 10.1111/jcmm.18164 (PMC10915828; doi:10.1111/jcmm.18164)
Supplement: Supplementary file 5 — Table S2. [file JCMM-28-e18164-s003.doc]

Table SⅡ. The information of the antibodies used in our study.

| Antibodies | Source | Identifier | Dilution ratio |
| --- | --- | --- | --- |
| Anti-UBA52 for western blot | Abcam | Cat#ab109227 | 1:1000 |
| Anti-UBA52 for immunohistochemistry | Affinity | Cat#AF0289 | 1:200 |
| Anti-EMC6 for western blot and immunoprecipitation | Abcam | Cat#ab84902 | 1:1000  1:200 |
| Anti-EMC6 for immunohistochemistry | Bioss | Cat#bs-16577R | 1:200 |
| Anti-P62 for western blot | Proteintech | Cat#18420-1-AP | 1:1000 |
| Anti-P62 for immunohistochemistry | Servicebio | Cat#Q13501 | 1:200 |
| Anti-LC3 for western blot | Proteintech | Cat#14600-1-AP | 1:1000 |
| Anti-beta Actin for western blot | Proteintech | Cat#20536-1-AP | 1:1000 |
| Anti-HA for immunoprecipitation | Abmart | Cat#M20003 | 1:100 |
| Anti-FLAG for immunoprecipitation | Abmart | Cat#M20008 | 1:100 |
| Goat anti-mouse IgG (H＆L) for western blot | Zenbio | Cat#511103 | 1:5000 |
| Goat anti-rabbit IgG (H＆L) for western blot | Zenbio | Cat#511203 | 1:5000 |
