# Supplementary material for: Inhibition of UBA52 induces autophagy via EMC6 to suppress hepatocellular carcinoma tumorigenesis and progression
Source: J Cell Mol Med. 2024 Mar 6;28(6):e18164. doi: 10.1111/jcmm.18164 (PMC10915828; doi:10.1111/jcmm.18164)
Supplement: Supplementary file 6 — Table S3. [file JCMM-28-e18164-s006.doc]

Table SⅢ. The plasmid sequences were the following.

| Name | Sequence 5’to3’ |
| --- | --- |
| shUBA52#1 | 5’-CTTGCCCAGAAATACAACT-3’ |
| shUBA52#2 | 5’-GCAAGAAGAAGTGTGGTCA-3’ |
| shUBA52#3 | 5’-GACCAGCAGCGTCTGATATTT-3’ |
| shUBA52#4 | 5’-GCCCAGTGACACCATTGAGAA-3’ |
| shUBA52#5 | 5’-GCAAGAAGAAGTGTGGTCACA-3’ |
| shNC | 5’-TTCTCCGAACGTGTCACGT-3’ |
| siUBA52#1 | 5’-CUUGCCCAGAAAUACAACUTT-3’ |
| siUBA52#2 | 5’-GUCUGAUAUUUGCCGGCAATT-3’ |
| siUBA52#3 | 5’-GCAAGAAGAAGUGUGGUCATT-3’ |
| siEMC6#1 | 5’-GCCUCUACGGCUUCAUCUUTT-3’ |
| siEMC6#2 | 5’-GUUCUGGACGUUCCUCUACTT-3’ |
| siEMC6#3 | 5’-AGGAGGUGGAACAAAUAUUTT-3’ |
| siNC | 5’-UUCUCCGAACGUGUCACGUTT-3’ |
