# Supplementary material for: Inhibition of UBA52 induces autophagy via EMC6 to suppress hepatocellular carcinoma tumorigenesis and progression
Source: J Cell Mol Med. 2024 Mar 6;28(6):e18164. doi: 10.1111/jcmm.18164 (PMC10915828; doi:10.1111/jcmm.18164)
Supplement: Supplementary file 7 — Table S4. [file JCMM-28-e18164-s002.doc]

Table SⅣ. The sequences of primers used were the following.

| Genes | Forward | Reverse |
| --- | --- | --- |
| UBA52 | AAGACAAGGAGGGTATCCCAC | TGTTGTAGTCTGAGAGAGTGCG |
| EMC6 | GCCGCCGTCCTGGATTATT | GAGGCGAGCAGGTAGAAGAT |
| GAPDH | ACCACCCTGTTGCTGTAGCCAA | GTCTCCTCTGACTTCAACAGCG |
