# Supplementary material for: Inhibition of UBA52 induces autophagy via EMC6 to suppress hepatocellular carcinoma tumorigenesis and progression
Source: J Cell Mol Med. 2024 Mar 6;28(6):e18164. doi: 10.1111/jcmm.18164 (PMC10915828; doi:10.1111/jcmm.18164)
Supplement: Supplementary file 8 — Table S5. [file JCMM-28-e18164-s001.doc]

Table SⅤ. The differentially expressed autophagy related genes between siNC and siUBA52 groups in Huh7 cells.

| ID | Gene Symbol | FDR | Log2FC | Regulated |
| --- | --- | --- | --- | --- |
| ENSG00000047056 | WDR37 | 0.000396873 | -0.595742917 | down |
| ENSG00000113273 | ARSB | 1.45E-10 | -0.823798152 | down |
| ENSG00000125703 | ATG4C | 2.19E-06 | 0.679099312 | up |
| ENSG00000127774 | EMC6 | 4.33E-07 | 1.137582222 | up |
| ENSG00000130734 | ATG4D | 0.042471854 | -0.75752054 | down |
| ENSG00000165861 | ZFYVE1 | 0.002625119 | -0.613492547 | down |
| ENSG00000168209 | DDIT4 | 0.000393534 | -0.60328524 | down |

ID, Identity; FDR, False discovery rate; Log2FC, Log2 fold change.
