# Supplementary material for: Inhibition of UBA52 induces autophagy via EMC6 to suppress hepatocellular carcinoma tumorigenesis and progression
Source: J Cell Mol Med. 2024 Mar 6;28(6):e18164. doi: 10.1111/jcmm.18164 (PMC10915828; doi:10.1111/jcmm.18164)
Supplement: Supplementary file 9 — Table S6. [file JCMM-28-e18164-s007.doc]

Table SⅥ. The first 10 differential gene enrichment pathways that are correlated with EMC6 in the TCGA-LIHC database.

| Name | ES | NES | NOM p-val | FDR q-val |
| --- | --- | --- | --- | --- |
| Aminoacyl tRNA biosynthesis | 0.78 | 1.99 | 0.000 | 0.037 |
| Wnt signaling pathway | 0.62 | 1.95 | 0.000 | 0.037 |
| Regulation of autophagy | 0.66 | 1.94 | 0.000 | 0.027 |
| RNA degradation | 0.73 | 1.93 | 0.000 | 0.024 |
| One carbon pool by folate | 0.71 | 1.92 | 0.000 | 0.023 |
| Ubiquitin mediated proteolysis | 0.69 | 1.91 | 0.000 | 0.022 |
| Endocytosis | 0.63 | 1.91 | 0.000 | 0.020 |
| Insulin signaling pathway | 0.58 | 1.88 | 0.000 | 0.029 |
| Chronic myeloid leukemia | 0.67 | 1.87 | 0.000 | 0.026 |
| Pyrimidine metabolism | 0.61 | 1.87 | 0.000 | 0.024 |

ES, Enrichment Score; NES, Normalized Enrichment Score; NOM p-val, Nominal p value; FDR q-val, False discovery rate.
