# Supplementary material for: Inhibition of UBA52 induces autophagy via EMC6 to suppress hepatocellular carcinoma tumorigenesis and progression
Source: J Cell Mol Med. 2024 Mar 6;28(6):e18164. doi: 10.1111/jcmm.18164 (PMC10915828; doi:10.1111/jcmm.18164)
Supplement: Supplementary file 10 — Data S1. [file JCMM-28-e18164-s010.docx]

**Figure Legend**

**Figure S1**. Western blot analysis results showing the protein level of UBA52 in HepG2 (A) and Huh7 (B) cells infected with the series of shUBA52 and shNC lentiviruses. WT, wild type; shNC, negative control short hairpin RNA; shUBA52, short hairpin RNA targeting UBA52.

**Figure S2**. Overexpression of UBA52 promotes the proliferation and migration of HCC cells. (A) Western blot analysis results showing the protein level of UBA52 in Hep3B cells infected with LV-UBA52 and LV-NC. (B) qRT‒PCR analysis results showing the mRNA level of UBA52 in Hep3B cells infected with LV-UBA52 and LV-NC. (C) A CCK-8 assay was used to evaluate the proliferation ability of Hep3B cells in the shNC group and shUBA52 group. (D) A colony formation assay was used to evaluate the proliferation ability of Hep3B cells in the shNC group and shUBA52 group. The bar graph on the right shows the quantification of clonogenicity (n = 3, mean ± SD). (E) A wound healing assay was performed to evaluate the migration ability of Hep3B cells in the shNC group and shUBA52 group. Scale bar = 25 μm. (F) A Transwell assay was performed to evaluate the migration ability of Hep3B cells in each group. Scale bar = 10 μm. **P* < 0.05, ***P* < 0.01, ****P* < 0.001. OD450, optical density at 450 nm values; LV-, lentivirus.

**Figure S3**. The expression of EMC6 is positively correlated with autophagy. (A) GSEA results showing the positive correlation between EMC6 expression and autophagy in HCC. (B, C) Western blot analysis results showing the protein level of EMC6 in HepG2 (B) and Huh7 (C) cells. (D, E) Western blot analysis results showing the protein levels of EMC6, P62, LC3I and LC3II in HepG2 (D) and Huh7 (E) cells transfected with siEMC6 and siNC. **P* < 0.05, ***P* < 0.01, ****P* < 0.001. siNC, negative control small interfering RNA; siEMC6, small interfering RNA against EMC6.
